# Letrozole versus coenzyme Q10 plus clomiphene citrate for women with polycystic ovarian syndrome: An efficacy and safety analysis

**DOI:** 10.17305/bb.2025.11386

**Published:** 2025-04-23

**Authors:** Juan Hou, Qingfen Chang

**Affiliations:** 1 Department of Gynecology and Obstetrics, Xi’an People’s Hospital (Xi’an Fourth Hospital), Xi’an City, China

**Keywords:** Clomiphene citrate, coenzyme Q10, letrozole, polycystic ovarian syndrome, PCOS, pregnancy, rotterdam criteria

## Abstract

Clomiphene citrate is a well-established treatment for Polycystic Ovarian Syndrome (PCOS) but has poor efficacy and adverse effects. Coenzyme Q10 supplementation improves mitochondrial function. Letrozole has been reported to be effective with fewer adverse effects but is not approved for PCOS by the USFDA. This is a retrospective study in women diagnosed with PCOS to assess treatment with either 2.5 mg/day letrozole (LO cohort, *n* ═ 103) for 5 days per cycle (for 9 cycles). The QC group received additional doses of 50 mg coenzyme Q10 three times daily (QC cohort, *n* ═ 123). A third group received only 100 mg/day clomiphene citrate (CC cohort, *n* ═ 155) from the second day of the menstrual cycle for 5 days. After treatment, the duration of the menstrual cycle decreased across all cohorts (*P* < 0.001 for all), with a smaller reduction observed in the LTZ cohort compared to the QC and CC cohorts (*P* < 0.05 for all). The number of conceived pregnancies in the LTZ cohort (*P* < 0.0001) and the CC + QC cohort (*P* < 0.0001) was significantly higher than in the CC only group. Similarly, conception was higher in the CC + Q10 group than in the CC only group (*P* < 0.0001 for both groups). Letrozole versus clomiphene citrate plus coenzyme Q10 showed similar efficacy in achieving pregnancy in women with PCOS.

## Introduction

Polycystic ovarian syndrome (PCOS) is the most common cause of oligomenorrhea (infrequent periods) and amenorrhea (absence of periods), affecting approximately 4%–8% of women of reproductive age worldwide [1]. Many women with oligomenorrhea or amenorrhea experience subfertility [2]. While most eventually conceive naturally, the process often takes longer, and only a small percentage require fertility treatment [3]. The pathogenesis of PCOS is complex and not fully understood. However, it is thought to be associated with abnormal levels of pituitary, luteinizing, and androgen hormones [4]. Hormone levels are generally higher during pregnancy [5]. Several treatment options are available for PCOS. One well-established therapy is clomiphene citrate (CC), a selective estrogen receptor modulator that acts on the hypothalamus to promote ovulation [1, 6]. However, CC has limited efficacy [7] and may cause adverse effects, including negative changes to cervical mucus and the endometrium [3]. In women resistant to CC, insulin-sensitizing agents such as metformin can improve its effectiveness [8]. Oxidative stress markers are elevated in women with PCOS [9]. Coenzyme Q10, an antioxidant involved in the electron transport chain [10], has been shown to improve mitochondrial function [11]. Although an optimal dose for PCOS has not been established, a typical prescription ranges from 100–200 mg [12]. Coenzyme Q10 is primarily produced through the action of adenosine triphosphate [1]. Letrozole (LO), an aromatase inhibitor, has been recently introduced as a treatment option [3]. It has been reported to lack anti-estrogenic effects on the endometrium [6] and enhances the secretion of gonadotropin-releasing hormone [2]. With a half-life of 48 h and fewer adverse effects, particularly on the cervix, LO has become the most commonly prescribed medication for women with PCOS [13, 14]. However, it is not approved by the US Food and Drug Administration (USFDA) for use in PCOS due to risks, such as ovarian hyperstimulation syndrome and multiple pregnancies [6, 15]. The objective of this retrospective analysis was to evaluate the efficacy (defined as the ability to conceive after treatment) and safety of LO in women with PCOS, compared with those who received either co-enzyme Q10 plus CC or CC alone, in a Chinese clinical setting.

## Materials and methods

### Study design, setting, and period

A retrospective analysis was conducted using data collected from hospital records at Xi’an People’s Hospital (Xi’an Fourth Hospital) in Xi’an City, Shanxi Province, China, covering the period from January 14, 2020 to March 1, 2023.

### Inclusion criteria

Women with PCOS (according to the Rotterdam criteria 2003) were included in the study.

### Exclusion criteria

Women with incomplete hospital data or left treatments (for any reason for example, intolerable symptoms) were excluded from the analyses. Women who had difficulty in conceiving pregnancies but did not have PCOS were excluded from the study.

### Sample size calculations

This study was based on the assumption that at least 30% of women receiving treatment for PCOS would conceive (regardless of treatment cycles, effect size, primary objective, or institutional protocol, which has not yet been published) [16]. Based on this assumption, with a power of 80% and an α level of 0.05, the required sample size for each arm was 100 participants [6].

### Diagnosis of PCOS

The presence of any two out of the three “Rotterdam criteria 2003” (Table 1) was considered diagnostic for PCOS [3, 17]. In the gynecology and obstetrics departments of the institutions, gynecologists and obstetricians evaluated the criteria for PCOS in women.

**Table 1:** Rotterdam criteria 2003 used in the study for polycystic ovarian syndrome

**Number**	**Criteria**
1	Absence of ovulation (anovulation) or infrequent ovulation (oligo-ovulation)
2	Hyperandrogenism (high level of androgen)
3	Polycystic ovary

### Cohorts

A total of 103 women received LO (2.5 mg) daily for five days starting on the second day of their menstrual cycle (LO cohort). Another 123 women received 50 mg of coenzyme Q10 three times daily along with 100 mg of CC once daily for five days from the second day of their cycle (QC cohort). A further 155 women received 100 mg of CC once daily for five days from the second day of their cycle (CC cohort). Cohort assignment was at the discretion of the attending obstetricians. Each patient received up to nine treatment cycles—exceeding the manufacturer’s recommendation of a maximum of six cycles for CC, due to a potential increased risk of ovarian cancer [18]. While NICE guidelines also recommend a limit of six cycles, they allow for the possibility of extending treatment to 12 cycles in certain cases. However, CC (Clomid) should not be used for more than six cycles in a patient’s lifetime, as the risk of ovarian cancer appears to increase slightly if used for more than 12 cycles [19].

### Outcome measures

#### Demographical and clinical conditions

Demographical and clinical conditions of women were collected from hospital records and analyzed.

#### Conceive of pregnancies (primary outcomes)

It was confirmed by ultrasonography.

#### Secondary outcomes

Secondary outcomes, for examples, pregnancy outcomes, pregnancy complications, and neonatal complications were evaluated and analyzed.

### Clinical benefits for treatments of PCOS (tertiary outcomes)

The clinical benefits of treatments for PCOS in women were evaluated using beneficial scores. These scores were calculated based on the risk of under-treatment, as defined in Equation (1). The risk of under-treatment was determined using the incidence of clinical pregnancies in women who received treatment, as shown in Equation (2). The beneficial score represents the area above the treatment response curve, while the working area corresponds to the area under this curve. For all evaluated treatments, 30% of clinical pregnancies following PCOS treatment were used as the reference standard [20]
(1)
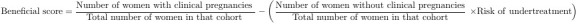

(2)
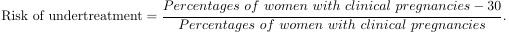


### Ethical statement

The protocols for the established study (XPHUB15pt, dated December 14, 2020) were prepared by the authors and approved by the Xi’an People’s Hospital Review Board. The study complies with Chinese laws and adheres to the 2008 version of the Declaration of Helsinki. The requirement for informed consent was waived by the Xi’an People’s Hospital Review Board.

### Statistical analysis

InStat 3.01 (GraphPad Software, San Diego, CA, USA) was used for statistical analysis. Non-normal continuous variables are presented as medians with interquartile ranges (Q3–Q1) in parentheses; categorical variables as frequencies with percentages in parentheses; and normal continuous variables as means ± standard deviations (SD). The normality of continuous variables was assessed using the Kolmogorov–Smirnov test. Fisher’s exact test or the chi-square (χ^2^) test with Yates’ correction was used for the analysis of categorical variables. One-way analysis of variance (ANOVA) was used for the analysis of normal continuous variables. For non-normal continuous variables, statistical analysis was conducted using the Kruskal–Wallis test (nonparametric ANOVA; between cohorts), and the Friedman test or the Wilcoxon matched-pairs signed-ranks test (within cohorts). Results were considered statistically significant at a *P* value of less than 0.05.

## Results

### Study population

**Figure 1.**
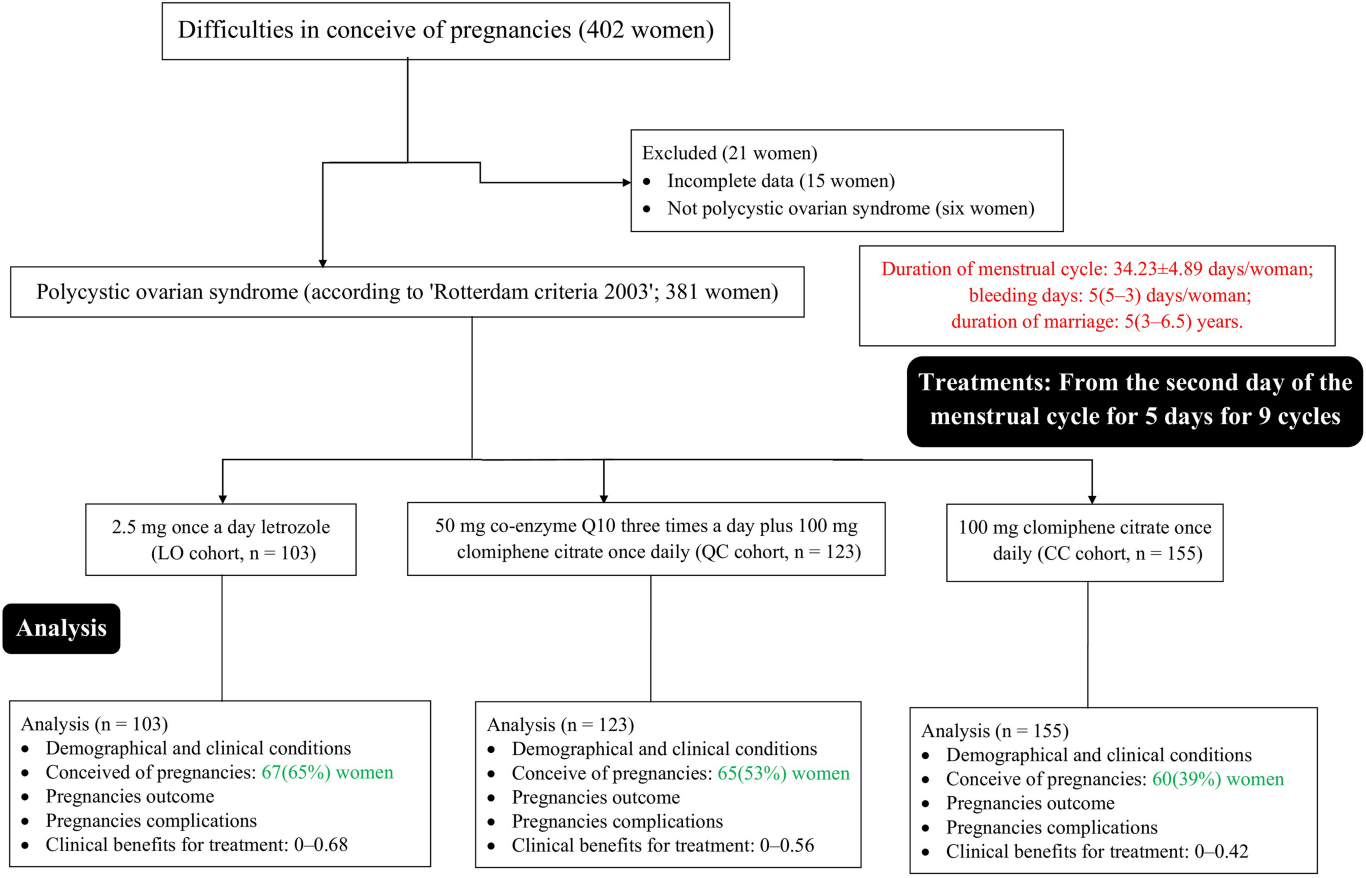
**Flow diagram of the study.** The red color indicates worse parameters. The green color indicates better variables.

Between January 14, 2020 and March 1, 2023, a total of 402 women with difficulties conceiving were seen at Xi’an People’s Hospital (Xi’an Fourth Hospital), Xi’an City, Shanxi Province, China, as well as at affiliated referring hospitals. Of these, complete medical records were unavailable for 15 women, and six women did not meet the diagnostic criteria for PCOS based on the Rotterdam Criteria (2003). As a result, these 21 women were excluded from the study. The final analysis included data on treatment and follow-up for 381 women with confirmed PCOS, according to the Rotterdam Criteria (2003). A flow diagram illustrating this retrospective analysis is presented in Figure 1.

### Outcome measures

#### Demographical and clinical conditions

The median age of the women was 26 years (range: 24–30 years). A total of 90% of participants were Han Chinese. The median duration of marriage was five years (range: 3–6.5 years), and the median body mass index (BMI) was 24.5 kg/m^2^ (range: 23–26 kg/m^2^). There were no significant differences among cohorts in demographic, anthropometric, or social parameters before the start of treatment (*P* > 0.05 for all). Detailed demographic, anthropometric, and social data are presented in Table 2 (blood pressure data were unavailable). Prior to treatment, the women had a mean menstrual cycle duration of 34.23 ± 4.89 days and a median of five days (range: 3–5 days) of menstrual bleeding. No significant differences in clinical conditions were observed among the cohorts before the start of treatment. Detailed clinical data are presented in Table 3.

**Table 2:** Demographical, anthropological, and social parameters of women before the start of treatment

**Parameters**	**Cohorts**					
	**LO**	**QC**	**CC**			
**Treatments**	**Letrozole**	**Co-enzyme Q10 + clomiphene citrate**	**Clomiphene citrate**	**Comparisons**		
Numbers of women	103	123	155	*P* value	Df	Test value
Age (years)	26 (28–25)	26 (27–25)	26 (27–25)	0.0806 (Kruskal–Wallis’ test)	N/A	5.037
*Ethnicity*						
Han Chinese	94 (91)	111 (90)	141 (89)	0.9857 (χ^2^-test for Independence)	6	0.9964
Mongolian	7 (7)	10 (8)	12 (8)			
Tibetan	1 (1)	1 (1)	3 (2)			
Uyghur Muslim	1 (1)	1 (1)	1 (1)			
Duration of marriage (years)	5 (5.5–4.5)	5 (5.5–4)	4.5 (5–4)	0.0957 (Kruskal–Wallis’ test)	N/A	4.692
Body mass index (kg/m^2^)	24 (25–23.5)	24 (25.5–24)	24.5 (24.5–23.5)	0.4901 (Kruskal–Wallis’ test)	N/A	1.426

Non-normal continuous and categorical variables are depicted as median with Q3–Q1 in parenthesis and frequencies with percentages in parenthesis. Df: Degree of freedom; N/A: Not applicable; CC: Clomiphene citrate; LO: Letrozole; χ^2^: Chi-squared. Test value (Kruskal–Wallis’ Statistic for Kruskal–Wallis’ test, chi-square value for chi-square test). All results were considered significant if the *P* value was less than 0.05.

**Table 3:** Clinical conditions of women before the start of treatment

**Parameters**	**Cohorts**					
	**LO**	**QC**	**CC**			
**Treatments**	**Letrozole**	**Co-enzyme Q10 + clomiphene citrate**	**Clomiphene citrate**	**Comparisons**		
Numbers of women	103	123	155	*P* value	Df	Test value
Duration of menstrual cycle (days)	33.38 ± 4.83	34.34 ± 4.84	34.7 ± 4.93	0.0982 (one-way ANOVA)	380	2.335
Bleeding days	5 (5–3)	5 (5–3)	5 (5–3)	0.1286 (Kruskal–Wallis’ test)	N/A	4.102

Non-normal continuous and normal continuous variables are depicted as median with Q3–Q1 in parenthesis and mean ± standard deviation (SD). Df: Degree of freedom; N/A: Not applicable; ANOVA: Analysis of variance; CC: Clomiphene citrate; LO: Letrozole. Test value (Kruskal–Wallis’ statistic for Kruskal–Wallis’ test, *F* value for one-way ANOVA). All results were considered significant if the *P* value was less than 0.05.

**Table 4:** Clinical conditions of women before and after the start of treatments

**Parameters**	**Cohorts**											
	**CC**				**QC**				**LO**			
**Treatments**	**Clomiphene citrate**				**Co-enzyme Q10 + clomiphene citrate**				**Letrozole**			
**Level**	**BT**	**AT**	***P* value**	**Test value**	**BT**	**AT**	***P* value**	**Test value**	**BT**	**AT**	***P* value**	**Test value**
Numbers of women	155	155			123	123			103	103		
Duration of menstrual cycle (days)	33 (38–29)	28 (29–28)	<0.001 (Friedman test)	93	34 (39–30)	28 (30–28)	<0.001 (Friedman test)	131	35 (39–31)	30 (33–28)	<0.001 (Friedman test)	145
Bleeding days	5 (5–3)	4 (5–3)	0.8536 (Wilcoxon test)	N/A	5 (5–3)	5 (5–4)	0.2078 (Wilcoxon test)	N/A	5 (5–3)	4 (4–4)	<0.001 (Friedman test)	24
Conceive of pregnancies	0 (0)	60 (39)	<0.0001 (Fisher’ exact test)	0	0 (0)	65 (53)	<0.0001 (Fisher’ exact test)	0	0 (0)	67 (65)	<0.0001 (Fisher’ exact test)	0

Non-normal continuous and categorical variables are depicted as median. with Q3–Q1 in parenthesis and frequencies with percentages in parenthesis. N/A: Not applicable; BT: Before treatment; AT: After treatment; CC: Clomiphene citrate; LO: Letrozole. *P* value between BT and AT. Test value (Kruskal–Wallis’ statistic for Kruskal–Wallis’ test, relative risk for Fisher’s exact test, Friedman statistic for Friedman test). 95% Confidence Interval: -Infinity to Infinity (using the approximation of Katz.) for all Fisher’s exact tests. All results were considered significant if the *P* value was less than 0.05.

#### Primary outcomes

After treatment, the duration of the menstrual cycle decreased across all cohorts compared to before treatment. Bleeding days also decreased, but only in the LO cohort relative to pre-treatment conditions. Post-treatment, the LO cohort had a significantly shorter menstrual cycle compared to the QC and CC cohorts (*P* < 0.001, Kruskal–Wallis statistic: 65). Similarly, the QC cohort showed a shorter cycle than the CC cohort (*P* < 0.05, Kruskal–Wallis statistic: 65). Bleeding days post-treatment were significantly fewer in the LO cohort compared to the CC (*P* < 0.001) and QC (*P* < 0.05) cohorts (Kruskal–Wallis statistic: 16), while there was no significant difference between the QC and CC cohorts (*P* > 0.05). In terms of pregnancy outcomes after treatment, 60 women (39%) conceived in the CC cohort, 65 (53%) in the QC cohort, and 67 (65%) in the LO cohort. More women conceived in the LO cohort than in the QC and CC cohorts, and more women conceived in the QC cohort than in the CC cohort. Outcome details are provided in Table 4.

#### Secondary outcomes

Dizziness, nausea, and headache were reported in nearly all women undergoing treatment. In addition to these pregnancy-related symptoms, most pregnancy and neonatal complications were attributed to the anesthesia method and pain management used during delivery. These complications were not linked to the medications prescribed for PCOS and, therefore, were not included in the current study.

### Clinical benefits for treatments of PCOS

**Figure 2.**
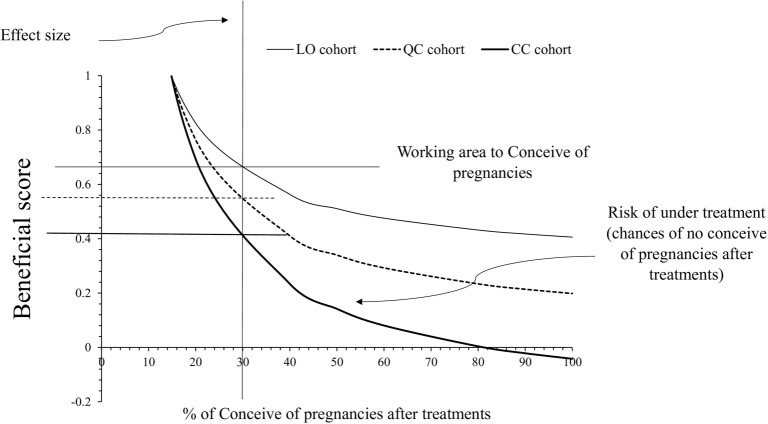
**Graphical presentation of clinical benefits of treatments.** LO: Letrozole; CC: Clomiphene citrate.

CC can result in pregnancy in up to 80% of women for whom it is prescribed. However, a combination of co-enzyme Q10 with CC, as well as LO alone, led to successful pregnancies in 100% of the women in whom these treatments were prescribed. The beneficial score ranges indicating the effective treatment window for achieving pregnancy were 0–0.68, 0–0.56, and 0–0.42 for the LO (letrozole only), QC (co-enzyme Q10 plus CC), and CC (clomiphene citrate only) cohorts, respectively. Beyond these thresholds—0.68 for LO, 0.56 for QC, and 0.42 for CC—there is a risk of undertreatment, meaning a reduced chance of conceiving after treatment. A graphical representation of the clinical benefits of these PCOS treatments is shown in Figure 2, while detailed data on the clinical benefits can be found in Table 5. The results of the assumptions test are presented in Table 6.

**Table 5:** Clinical benefits of treatments of polycystic ovarian syndrome

**%Conceive of pregnancies**	**Beneficial score of cohorts**		
	**LO**	**QC**	**CC**
	**Letrozole**	**Co-enzyme Q10 + clomiphene citrate**	**Clomiphene citrate**
Numbers of women	103	123	155
Women with conceive of pregnancies	67	65	60
Women without conceive of pregnancies	36	58	95
1	10.79	14.20	18.16
5	2.4	2.89	3.45
10	1.35	1.47	1.61
20	0.83	0.76	0.69
40	0.56	0.41	0.23
50	0.51	0.34	0.14
60	0.48	0.29	0.08
80	0.43	0.23	0.004
90	0.42	0.21	−0.02
100	0.41	0.2	−0.04
Working area (beneficial score)	0–0.68	0–0.56	0–0.42
Risk of under treatment (beneficial score)	>0.68	>0.56	>0.42

Effect size: A minimum of 30% of women who received treatments for polycystic ovarian syndrome would conceive pregnancies. CC: Clomiphene citrate; LO: Letrozole.

**Table 6:** Results of assumption tests

**Variables**	**Adopted test (with conclusions)**
*Categorical variables*	
2 × 2 tables	Chi-square test with Yate’s correction if the sample size was more than 50 and the individual sample was more than five otherwise Fisher exact test
Large tables	Chi-square test with independence
*Continuous variables*	
*Demographical and clinical parameters*	
Age (years)	All columns failed in the normality test; *P* values were 0.0169; <0.0001; 0.0003. Therefore, Kruskal–Wallis’ test (nonparametric ANOVA)
Duration of marriage (years)	All columns failed in the normality test; *P* values were 0.0004; 0.0009; 0.0001. Therefore, Kruskal–Wallis’ test (nonparametric ANOVA)
Body mass index (kg/m^2^)	All columns failed in the normality test; *P* values were <0.0001 for all. Therefore, Kruskal–Wallis’ test (nonparametric ANOVA)
Duration of menstrual cycle (days)	All columns were passed in the normality test; *P* values were 0.0762; 0.0697; 0.0844. Bartlett statistic (corrected) ═ 0.07037. The *P* value is 0.9654. Bartlett’s test suggests that the differences among the standard deviations (SD)s are not significant. Therefore, one-way ANOVA
Bleeding days	All columns failed in the normality test; *P* values were <0.0001 for all. Therefore, Kruskal–Wallis’ test
*Outcome measures*	
Duration of menstrual cycle (days) and bleeding days (within cohort)	At least one column failed in the normality test; *P* values were <0.05. Therefore, the Friedman test (nonparametric repeated measures ANOVA) or Wilcoxon matched-pairs signed-ranks test
Duration of the menstrual cycle (days) and bleeding days (between cohorts)	At least one column failed in the normality test; *P* values were <0.05. Therefore, Kruskal–Wallis’ test

ANOVA: Analysis of variance.

## Discussion

Participants had an average menstrual cycle duration of 34.23 ± 4.89 days per woman, with an average of 5 (5–3) bleeding days per woman before the start of treatments for PCOS. Irregular menstrual bleeding and cycles are common symptoms and signs of PCOS [8]. The clinical characteristics observed in women in this study are consistent with those reported in randomized trials [1, 7, 10], crossover studies [6, 13], and prospective studies [21]. Chinese women are generally aware of PCOS, and those affected require appropriate treatment. More women in the QC cohort conceived pregnancies compared to the CC cohort. Additionally, the duration of the menstrual cycle and the number of bleeding days improved in the QC cohort relative to the CC cohort. Co-enzyme Q10 appears to enhance the likelihood of pregnancy by improving menstrual irregularities [1]. Diets high in protein and low in carbohydrates can worsen conditions in women with PCOS [22]. The outcome measures related to co-enzyme Q10 in this study align with findings from randomized trials [1] and prospective studies [21]. Combining co-enzyme Q10 with CC may improve the chances of conception in Chinese women with PCOS. However, fewer women in the QC cohort conceived pregnancies compared to the LO cohort. Furthermore, menstrual cycle duration and bleeding days did not improve in the QC cohort relative to the LO cohort. The benefits of co-enzyme Q10 may not be significantly enhanced when combined with CC in ovulation induction protocols [1]. The performance outcomes of co-enzyme Q10 in this study are consistent with those reported in randomized trials [1] and prospective studies [21]. Thus, the addition of co-enzyme Q10 to CC treatment for PCOS may not substantially improve ovulation induction outcomes.

At present, no study has evaluated the combined effects of co-enzyme Q10 and CC in the treatment of PCOS in Chinese populations. Given that the Chinese diet often includes foods enriched with co-enzyme Q10 [23], this study may offer meaningful clinical insights for the treatment of women with PCOS. However, the study has several limitations. Its retrospective design and lack of randomized trials limit the strength of its conclusions. Additionally, independent parameters of PCOS were not assessed. The small sample size also weakens the findings—larger cohorts would increase the reliability of the results. Furthermore, the power analysis appears insufficient, assuming a 30% baseline pregnancy rate without adequate justification or citation, which compromises the study’s statistical rigor. It is also important to note that this is not a study on infertility treatments for PCOS patients specifically. Although the medications studied are used in infertility treatments, their role in menstrual regulation for PCOS patients was not the focus here. Details of comparative studies on PCOS treatments in various settings are provided in Table 7.

**Table 7:** Comparative studies on the treatment of polycystic ovarian syndrome in different settings

**Study**	**Published year**	**Women ethnicity**	**Sample size (*N*; women)**	**Age (years)**	**Follow-up**
Randomized trial, Jamal et al. [1]	2023	Pakistani	136 (86 women each)	>18	12-months
Pragmatic randomized controlled trial, Chen et al. [2]	2024	Chinese	220	20–40	Three-months
Crossover study, Amer et al. [6]	2017	English	159 (79; 80)	18–39	12-months
In the randomized trial, Legro et al. [7]	2014	North American	750 (376; 374)	18–40	12-months
Randomized trial, Izhar et al. [10]	2022	Pakistani	133 (77; 72)	>18	12-months
Crossover study, Jirge and Patil [13]	2010	Indian	30	<39	12-months
Randomized controlled trial, Zhu et al. [14]	2024	Chinese	148 (74 each)	>18	24-months
Randomized controlled trial, Dai et al. [15]	2023	Chinese	174	18–40	28-weeks
Prospective study, Lakhmi et al. [21]	2018	Indian	40	18–25	12-months

## Conclusion

Chinese women are generally aware of PCOS, and those affected by it require appropriate treatment. The combination of co-enzyme Q10 and CC has been shown to improve the chances of conception in Chinese women with PCOS. However, adding co-enzyme Q10 to CC treatment may not significantly enhance ovulation induction parameters. Nonetheless, this study offers clinically relevant insights for the treatment of PCOS in the Chinese female population.

## Data Availability

The datasets used and analyzed during the current study are available from the corresponding author upon reasonable request.
